# Supplementary material for: BaZFP1, a C2H2 Subfamily Gene in Desiccation-Tolerant Moss Bryum argenteum, Positively Regulates Growth and Development in Arabidopsis and Mosses
Source: Int J Mol Sci. 2022 Oct 25;23(21):12894. doi: 10.3390/ijms232112894 (PMC9656138; doi:10.3390/ijms232112894)
Supplement: Supplementary file 1 [file ijms-23-12894-s001.zip › Table S2 List of primers overexpression vector construction.pdf]

Table S2 List of primers used for plant overexpression vector construction

**Primer name**	**Primers (5'to3')**
BaZFP1	F: ATGGAACTCTTTCTTTTGGC<br>R: CGATTGAGCAAATAG
pROKII	F: CAGGAAACAGCTATGAC<br>R: TGACCGGCAGCAAAATG
pROKII- BaZFP1 (Infusion)	F: CTCTAGAGGATCCCCGGGATGGAACTCTTTCTTTTGGC<br>R: TCGAGCTCGGTACCCGGGCGATTGAGCAAATAG
PBI121- BaZFP1-GFP (Infusion)	F: CAAGGGTCTAGACCCATGGAACTCTTTCTTTTGGC<br>R: TGGTGATGCATTCCCTTAATTGAGCAAATAGTTGAGTTCC
PBI121-GFP	F: GTCATAGCTGTTTCCTG<br>R: ACTGGCCGTCGTTTTAC
