# Supplementary material for: BaZFP1, a C2H2 Subfamily Gene in Desiccation-Tolerant Moss Bryum argenteum, Positively Regulates Growth and Development in Arabidopsis and Mosses
Source: Int J Mol Sci. 2022 Oct 25;23(21):12894. doi: 10.3390/ijms232112894 (PMC9656138; doi:10.3390/ijms232112894)
Supplement: Supplementary file 1 [file ijms-23-12894-s001.zip › Table S3 Primers for Arabidopsis real-time RT-PCR.pdf]

Table S3 Primers for *Arabidopsis* real-time RT-PCR

Gene name	Annotation	Primers (5'to3')
AtHLS1 (AT4G37580)	HOOKLESS 1	F: CTCTCGCTTGGGACTTTCGT<br>R: CAATCTCGACGCTCCACGTA
AtSAUR17 (AT4G09530)	SMALL AUXIN UPREGULATED RNA 17	F: TAACTATGCCGCACAAGGTCA<br>R: ACGTAAACGACGAAATGCCC
AtEIN3 (AT3G20770)	Ethylene insensitive 3	F: AAAGTCGGCGTTTTGACTGC<br>R: AGGTGGACATGACTCGGGAT
AtHY5 (AT5G11260)	ELONGATED HYPOCOTYL 5	F: TGTAAATCCCAAGTCCCGCT<br>R: TTGATGACCTCTCGCTGCTT
AtGAI (AT1G14920)	GIBBERELLIC ACID INSENSITIVE	F: CACGCCACAACTACAGGCTA<br>R: TGTGCCAACCCAACATGAGA
AtPIF4 (AT2G43010)	Phytochrome interacting factor 4	F: GACCGTTGGACCTAGCCATT<br>R: TGAGGAACTTGCGTTCGGAT
AtANT (AT4G37750)	AINTEGUMENTA	F: CTAGCTGCATCACTGGCTCT<br>R: TTGTCGTCGTCTCAAACCCA
AtAN3 (AT5G28640)	ANGUSTIFOLIA 3	F: CGAATGCGCCGAGAATCAAG<br>R: AATCATCCCACCACCAGCAG
AtGRF3 (AT2G36400)	Growth-regulating factor 3	F: TGACGATTGGCCTCGTTCTT<br>R: CTCCCATTGTTGTTGCTCCG
AtFT (At1g65480)	FLOWERING LOCUS T	F: TCTACAATCTCGGCCTTCCC<br>R: ATAGGCATCATCACCGTTCGT
AtLFY (AT5G61850)	Floral meristem identity control protein LEAFY	F: ATGGATAACGGCAACGGAGG<br>R: GCATTTTTCGCCACGGTCTT
AtFLC AT5G10140)	FLOWERING LOCUS C	F: GGCGACTTGAACCCAAACCT<br>R: GTTGCGACGTTTGGAGAAGG
AtTUA6 (AT4G14960)	Tubulin alpha-6	F: ACCCATCTCCACAGGTGTCT<br>R: CCTGAGAGACGAGACGGTTG
AtUBQ12 (AT1G55060)	Ubiquitin 12	F: TTGACGGGAAAGACCATCACG<br>R: AATCCGCTAAAGTGCGACC
