# Supplementary material for: BaZFP1, a C2H2 Subfamily Gene in Desiccation-Tolerant Moss Bryum argenteum, Positively Regulates Growth and Development in Arabidopsis and Mosses
Source: Int J Mol Sci. 2022 Oct 25;23(21):12894. doi: 10.3390/ijms232112894 (PMC9656138; doi:10.3390/ijms232112894)
Supplement: Supplementary file 1 [file ijms-23-12894-s001.zip › Table S4 Primers for moss real-time RT-PCR.pdf]

Table S4 Primers for *Bryum argenteum* and *Physcomitrella patens* real-time RT-PCR

Gene name	Annotation	Primers (5'to3')
PpDCL4 (EF670438.1)	Physcomitrella patens dicer-like 4 mRNA	F: TTGCTGCCAGGGTGGATAAG<br>R: ACACTGCTCCTGCTAACGAC
PpAPB4 (AB675592)	AINTEGUMENTA, PLETHORA and BABY BOOM	F: TCTCATCCTTGTTCCGGTGC<br>R: ATCCCAGCTTTCCGTGTCTG
PpSEC6 (Pp3c8_3920V3.1)	Core exocyst subunit	F: GCTGTTGCCTCTCCCTGATT<br>R: AGCCCTAGCTTGCTCTACCT
PpSTM1 (Pp3c1_27440: L)	STEM CELL-INDUCING FACTOR 1	F: ATACCGAGGAGTGCGACAAC<br>R: TCGTAGGGGAAATTGGTGCG
BaDCL4	Physcomitrella patens dicer-like 4 mRNA	F: CAACTGCTCCACATTGCTCG<br>R: GCAACAGCACAACACGACAT
BaAPB4	AINTEGUMENTA, PLETHORA and BABY BOOM	F: CACGCCACAACTACAGGCTA<br>R: CACAACTTCTTTCCGCCTGC
BaSEC6	Core exocyst subunit	F: ACTACGAGACGACATCCCCA<br>R: TTTCGGGAGGCAAGTGAGTC
BaSTM1	TEM CELL-INDUCING FACTOR 1	F: CCGTGCTTCGAGTGGTTTTG<br>R: TCAGAAACCTCCGCCATTCC
Pp-*Actin*	------	F: CGGAGAGGAAGTACAGTGTGTGGA<br>R: CTGGGCTCAATTCTAACGGCTGGT
Ba-Tublin	------	F: ATCACGGAGTTCCAGACCAA<br>R: CCACTGCCGCATTCACAT
