# Supplementary figures and images for: BaZFP1, a C2H2 Subfamily Gene in Desiccation-Tolerant Moss Bryum argenteum, Positively Regulates Growth and Development in Arabidopsis and Mosses
Source: Int J Mol Sci. 2022 Oct 25;23(21):12894. doi: 10.3390/ijms232112894 (PMC9656138; doi:10.3390/ijms232112894)

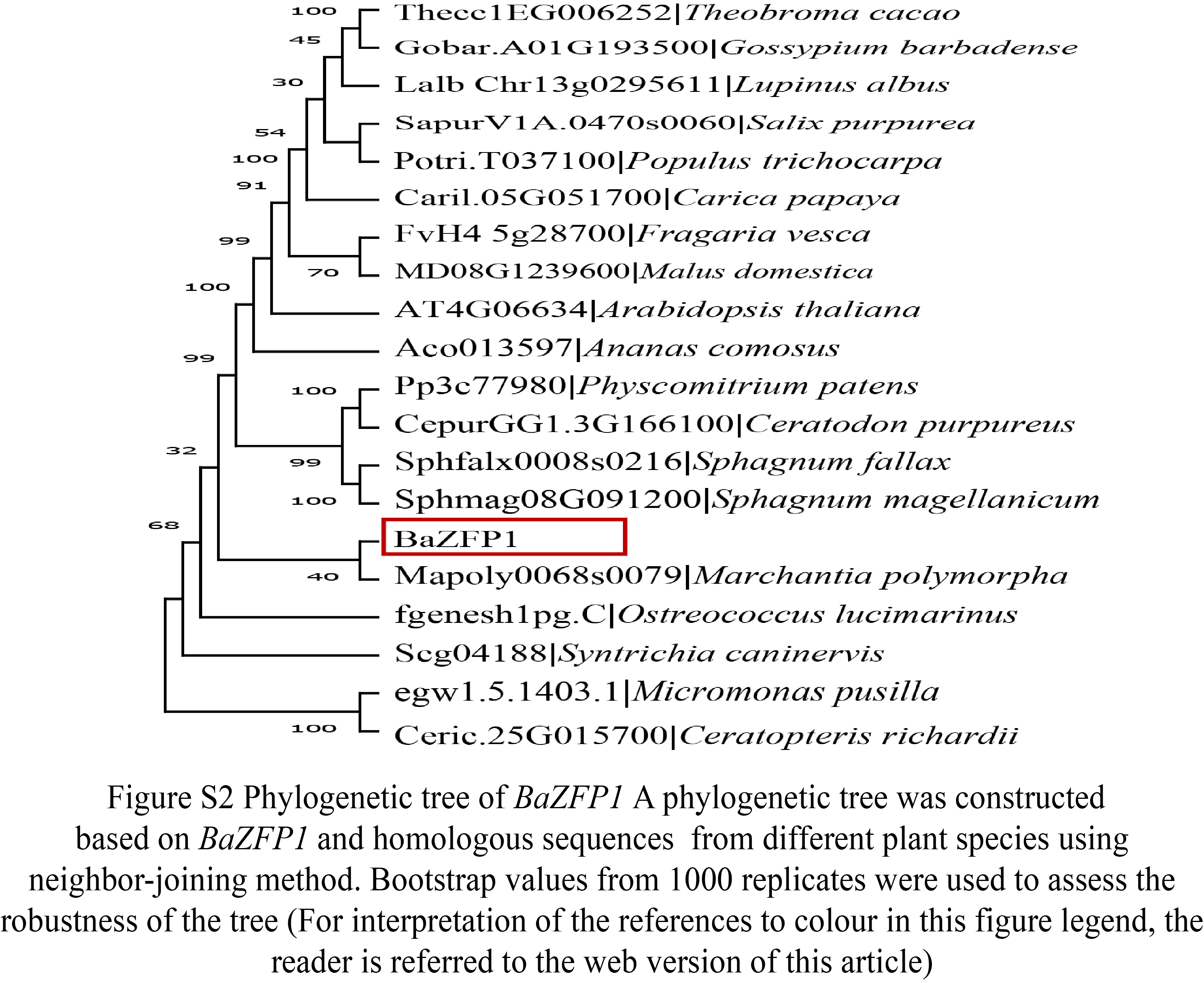

Supplement: Supplementary file 1 [file ijms-23-12894-s001.zip › Fig S2.tif]

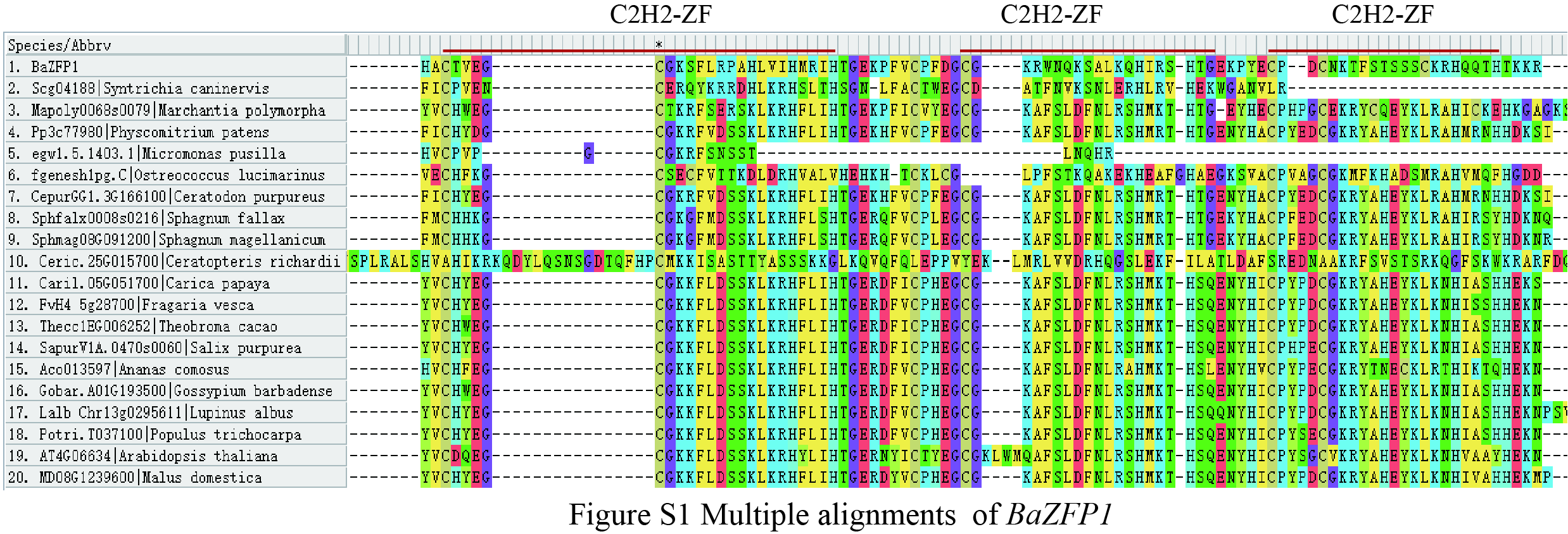

Supplement: Supplementary file 1 [file ijms-23-12894-s001.zip › Figure S1.tif]
